# Cutaneous clues to a fungal culprit: disseminated Blastomycosis presenting as inflammatory monoarthritis — a Case Report

**DOI:** 10.3389/fmed.2026.1704751

**Published:** 2026-02-18

**Authors:** Paddy Ssentongo, Sarah Akhtar, Zinaida Perciuleac, Courtland Kaye, Rekha Aley Cherian, Elizabeth Garrett, Shirley Albano-Aluquin, Thomas N. Helm, Leslie J. Parent

**Affiliations:** 1 Division of Infectious Diseases and Epidemiology, Department of Medicine, Penn State Hershey Medical Center, Hershey, PA, United States; 2 Division of General Internal Medicine, Department of Medicine, Penn State Hershey Medical Center, Hershey, PA, United States; 3 College of Medicine, Penn State University, Hershey, PA, United States; 4 Department of Radiology, Penn State Hershey Medical Center, Hershey, PA, United States; 5 Department of Pathology and Laboratory Medicine, Penn State Hershey Medical Center, Hershey, PA, United States; 6 Division of Rheumatology, Department of Medicine, Penn State Hershey Medical Center, Hershey, PA, United States; 7 Department of Dermatology, Penn State Hershey Medical Center, Hershey, PA, United States

**Keywords:** *Blastomyces dermatitidis*, cutaneous fungal infection, dimorphic fungi, disseminated blastomycosis, fungal septic arthritis, inflammatory monoarthritis, pulmonary nodules

## Abstract

Blastomycosis is a dimorphic fungal infection caused by *Blastomyces dermatitidis* and related species, classically presenting with pulmonary and cutaneous involvement. Musculoskeletal manifestations, particularly monoarthritis, are rare and diagnostically challenging. We present the case of a previously healthy 41-year-old man who developed nodular skin lesions, cough, pulmonary nodules and persistent left knee arthritis, initially thought to be due to pseudogout. The diagnosis of disseminated fungal infection was suggested by CT scan, skin biopsy, and confirmatory fungal culture that grew *Blastomyces dermatitidis.* This case highlights the atypical pattern of joint involvement, the need to expand the differentials as dictated by clinical signs, the role of multidisciplinary collaboration, and the diagnostic utility of skin biopsy and fungal culture.

## Introduction

Blastomycosis is an uncommon systemic mycosis caused by *Blastomyces dermatitidis*, a thermally dimorphic fungus endemic to the Ohio and Mississippi River valleys, the Great Lakes region, and parts of Canada (1, 2). Infection typically occurs via inhalation of aerosolized conidia, which undergo transformation into yeast in the host and produce primary pulmonary disease (3). Hematogenous dissemination occurs in approximately 20–50% of symptomatic cases, most commonly to the skin, bone, genitourinary tract, and central nervous system (1, 4).

Osteoarticular involvement is infrequent, reported in 10–25% of disseminated cases, and may involve synovial tissue, joint spaces, or adjacent bone (5–7). Diagnosis is often delayed because the clinical presentation can mimic other conditions such as crystal arthropathy, bacterial septic arthritis, or autoimmune rheumatologic disease (7). Early recognition and initiation of antifungal therapy are critical, as delays in diagnosis and treatment are associated with increased morbidity and mortality (8).

We describe an immunocompetent man with disseminated *Blastomyces dermatitidis* infection whose initial manifestation was inflammatory monoarthritis of the knee. Diagnosis required coordinated input from rheumatology, orthopedics, dermatology, infectious diseases, and microbiology, and was ultimately confirmed by culture and histopathologic examination of a cutaneous lesion. Initiation of antifungal therapy led to rapid improvement in both joint symptoms and cutaneous manifestations.

## Case

### Early course and outside evaluations

Approximately 6 weeks before admission to Penn State Health Milton S. Hershey Medical Center in Central Pennsylvania (Day −42), a 41-year-old previously healthy man developed progressive swelling and pain of the left knee, beginning 3 days after returning from a multi-day camping trip on an island in the Susquehanna River in central Pennsylvania, a large freshwater river system in the eastern United States. The patient had no significant past medical history, took no chronic medications, and had no known immunosuppressive conditions.

**Figure 1:**
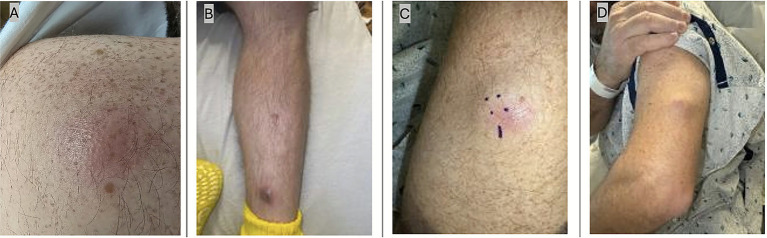
Representative cutaneous lesions at presentation. **(A)** Erythematous, tender nodule on the left upper shoulder. **(B)** Firm, dome-shaped nodule on the left anterior shin. **(C)** Erythematous nodule on the left shin marked for biopsy. **(D)** Subcutaneous nodule on the left upper arm, firm and mobile.

The pain was insidious in onset, without antecedent trauma, and gradually worsened over several days. Within the same week, he noticed the appearance of several nodular, tender “boils”—initially over the posterior aspect of the left shoulder, followed within days by similar lesions on the left upper arm and left anterior shin (Figure 1). He also reported a productive cough that had been ongoing for several months. This cough had been attributed to laryngopharyngeal reflux and empirically treated by an otolaryngologist with proton-pump inhibitors, montelukast, and lifestyle modification, without improvement. He denied dyspnea, pleuritic chest pain, or hemoptysis.

Five weeks prior to admission (Day −35), he sought evaluation at an outside facility because of worsening knee swelling and pain. Arthrocentesis of the left knee yielded approximately 60 mL of pink-tinged synovial fluid with a white-blood-cell count of ~13,000/mm^3^, a neutrophil predominance, and the presence of calcium pyrophosphate crystals. Rare coagulase-negative *Staphylococcus* grew in culture, which was regarded as a contaminant. Additional aerobic and anaerobic bacterial cultures were negative, and Lyme PCR was negative. Based on the presence of crystals, he was diagnosed with pseudogout and treated with colchicine and oral cephalexin for suspected overlying cellulitis at the outside facility.

Four weeks prior to admission (Day −28), in the setting of persistent symptoms, he received an intra-articular corticosteroid injection in the left knee, which produced transient symptomatic improvement for approximately 48 h before pain and swelling recurred. This procedure was performed at an outside orthopedic clinic prior to referral.

Three weeks prior to admission (Day −21), worsening pain prompted a repeat knee aspiration at another outside facility. Synovial fluid showed no organisms on Gram stain and no bacterial growth on culture. He was prescribed a course of oral clindamycin and naproxen. A cell count, differential, and crystal analysis were not documented from this aspiration. Despite these interventions, knee pain intensified, his range of motion decreased, and he required a walker for ambulation. During this period, his skin lesions persisted, and his cough remained unchanged.

Outside-hospital laboratory testing revealed leukocytosis (14 × 10^9^/L [reference range, 4–10 × 10^9^/L]) with neutrophil predominance (88% [40–70]), hemoglobin 15 g/dL (13.5–17.5), platelet count 302 × 10^9^/L (150–450), creatinine 1.07 mg/dL (0.6–1.3), calcium 9.3 mg/dL (8.5–10.5), and markedly elevated inflammatory markers (ESR > 100 mm/h [0–15], CRP > 100 mg/L [0–5]). Lyme serology was negative, consistent with prior PCR testing.

Given the persistence of symptoms despite multiple empirical treatments and procedures at outside facilities, the patient was referred for further evaluation.

### Hospital admission

On admission (Day 0), he had severe left knee pain that severely limited ambulation, along with night sweats and intermittent low-grade fevers up to 38 °C over the preceding several days. He did not report chills, rigors, weight loss, or recent upper-respiratory symptoms beyond his chronic cough.

Physical examination revealed a diffusely swollen and tender left knee with a moderate effusion and restriction in both flexion and extension, but without overlying erythema or warmth. Multiple discrete cutaneous nodules were noted:

- Erythematous, tender nodule on the posterior aspect of the left shoulder (Figure 1A)
- Left anterior shin: Two firm, dome-shaped nodules, each approximately 1.5–2 cm in diameter, erythematous at the periphery, and tender to palpation (Figures 1B,C)
- Left upper lateral arm: A single subcutaneous nodule measuring 2.0 cm, firm, mobile, mildly tender, without fluctuance (Figure 1D)
- Right lateral flank: One firm, non-tender, skin-colored nodule measuring 2.5 cm, without overlying erythema or drainage.

The nodules were well-circumscribed, non-ulcerated, and freely mobile, suggestive of dermal or subcutaneous involvement rather than deeper fascial extension.

Laboratory testing demonstrated WBC 20 × 10^9^/L (reference range, 4–10), hemoglobin 14 g/dL (13.5–17.5), creatinine 0.8 mg/dL (0.6–1.3), ESR 108 mm/h (0–15), and CRP 23 mg/L (0–5). Serum uric acid was 5.0 mg/dL (3.5–7.2). The remaining labs are shown in Supplementary Table 1. Given the markedly elevated inflammatory markers and prior inconclusive outpatient workups, broad infectious and inflammatory etiologies were considered.

**Figure 2:**
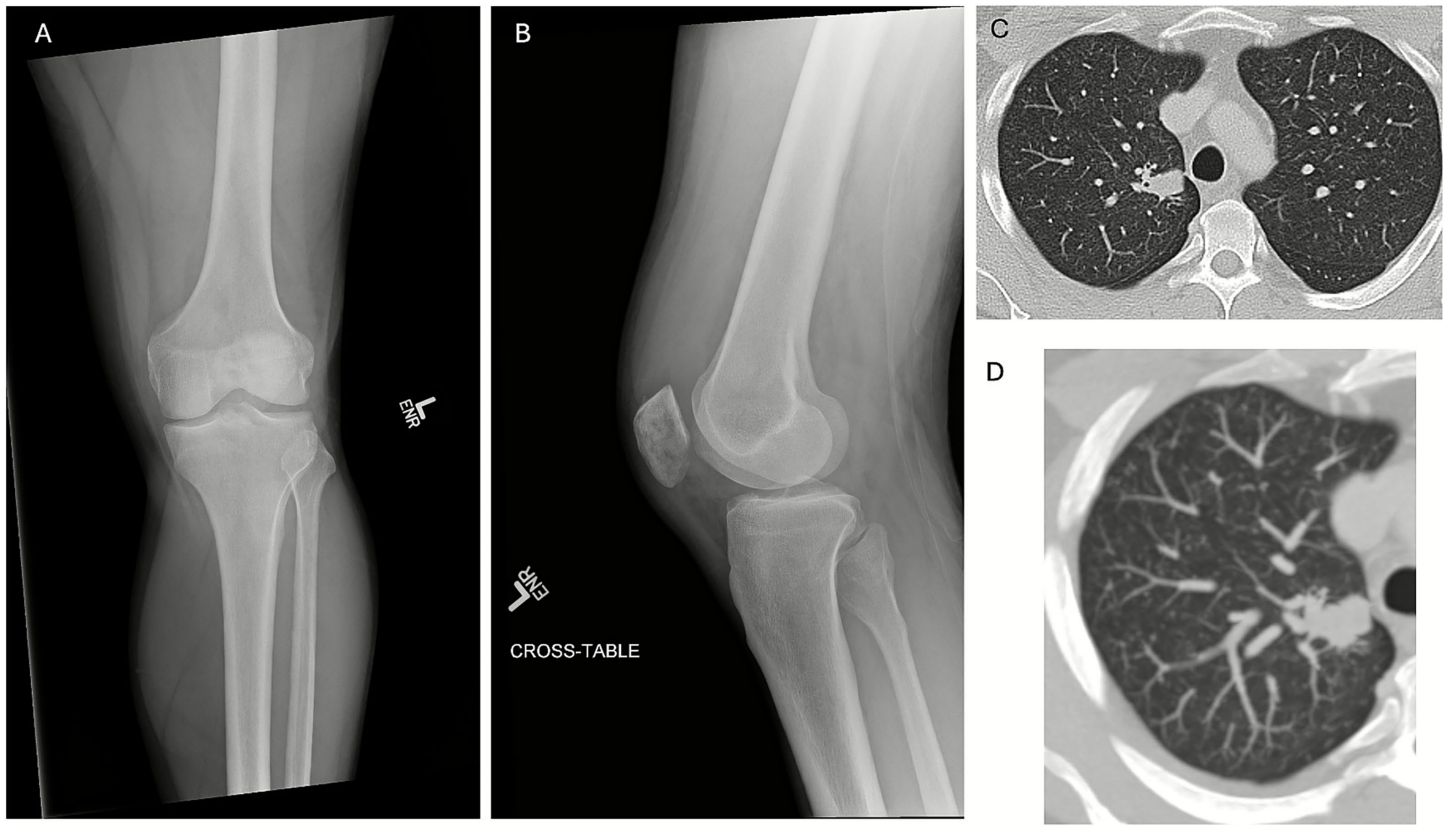
Radiographic and imaging findings of disseminated blastomycosis. **(A)** Anteroposterior and **(B)** lateral radiographs of the left knee demonstrate joint effusion with preserved joint spaces and patchy lucency in the patella. **(C)** Chest computed tomography showed focal consolidation in the apical segment of the right upper lobe and diffuse bilateral non-calcified miliary micronodules. **(D)** Maximum intensity projection (MIP) CT image better demonstrating diffuse micronodularity.

On-admission, radiographs of the left knee demonstrated a moderate joint effusion with patchy subchondral lucency along the inferior patella but preserved joint spaces (Figures 2A,B).

Arthrocentesis performed on the day of admission yielded 35 mL of thick yellow-green synovial fluid with a WBC count of 6,000/mm^3^ (92% neutrophils, 7% lymphocytes, 1% monocytes/macrophages). Crystal analysis was not initially requested. Gram stain revealed no organisms, and bacterial cultures remained sterile after prolonged incubation.

These findings were notable for a predominantly neutrophilic but only mildly inflammatory effusion, prompting consideration of atypical infectious or inflammatory etiologies. A comparison of synovial-fluid characteristics from the outside facility and our institution is summarized in Supplementary Table 2.

## Hospital course and diagnostic workup

Soon after admission, the patient was evaluated by Orthopedic Surgery, Rheumatology, Infectious Diseases, and Dermatology in close coordination.

The orthopedic assessment concluded that acute bacterial infection was unlikely, as the synovial WBC count (6,000/mm^3^) was well below the typical threshold for septic arthritis and Gram stain and cultures were negative. Non–weight-bearing precautions, knee immobilization, and further diagnostic evaluation were advised rather than surgical irrigation and debridement.

The rheumatology team considered inflammatory arthritis, with the leading possibilities being crystal-induced arthritis or vasculitis-associated monoarthritis. Given the absence of crystals on the most recent synovial-fluid analysis and the presence of multiple extra-articular skin nodules, the differential was broadened to include sarcoidosis, vasculitis, or reactive arthritis. Methylprednisolone was recommended at 40 mg intravenous daily and assessed regularly for therapeutic response. Autoimmune serologies were unremarkable: antinuclear antibody was negative, rheumatoid factor < 10 IU/mL (reference 0–14), and anti-CCP < 5 U/mL (0–19). Inflammatory markers remained markedly elevated (ESR 108 mm/h [0–15], CRP 23 mg/L [0–5]). Because the skin lesions resembled erythema nodosum, sarcoidosis was still considered, and a chest radiograph showed subtle diffuse interstitial prominence.

The infectious-diseases service raised concern for disseminated fungal infection given the subacute presentation, cutaneous nodules, chronic cough, and persistent monoarthritis unresponsive to antibacterial therapy. Fungal antigen testing and expanded microbiologic studies—including mycobacterial and fungal cultures from skin and synovial fluid—were requested. When urine *Histoplasma* antigen returned positive, this heightened suspicion for disseminated mycosis. Empiric liposomal amphotericin B was started while awaiting further results because of concern for moderate-to-severe disseminated infection.

Contrast-enhanced CT of the chest, abdomen, and pelvis showed numerous bilateral non-calcified pulmonary nodules in a diffuse miliary pattern and a focal right-upper-lobe consolidation compatible with hematogenous dissemination of fungal infection (Figures 2C,D). No lymphadenopathy, hepatosplenomegaly, or primary malignancy was evident. Brain MRI revealed no evidence of CNS blastomycosis. CT of the abdomen and pelvis showed no intra-abdominal abnormalities.

**Figure 3:**
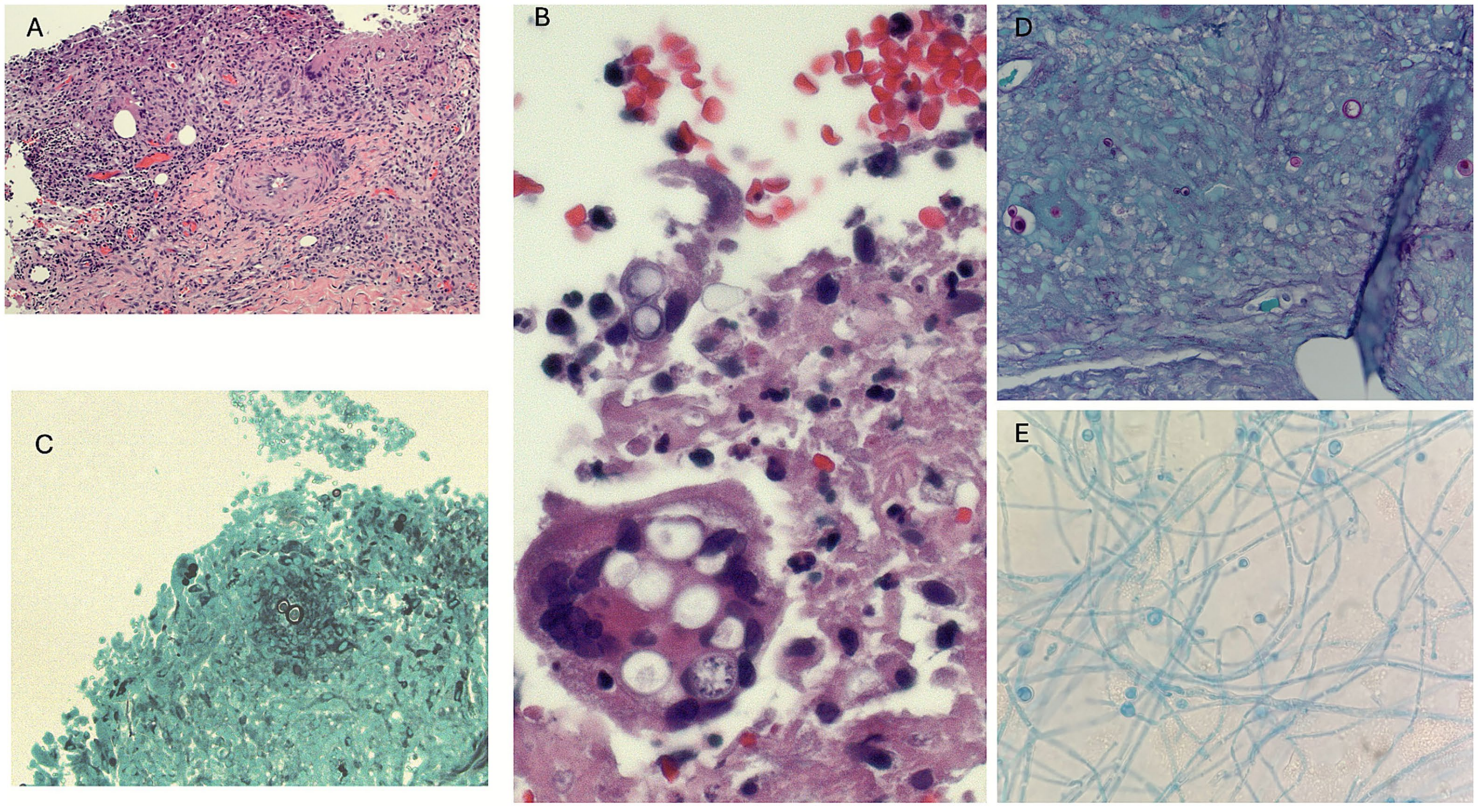
Histopathologic and microbiologic findings of disseminated blastomycosis. **(A)** Hematoxylin and eosin (H&E) stain of skin biopsy showing dermal granulomatous inflammation with mixed acute and chronic inflammatory infiltrates (original magnification 100x). **(B)** High-power H&E stain highlighting large, thick-walled yeast forms with broad-based budding (original magnification 400x). **(C)** Grocott methenamine silver (GMS) stain demonstrating yeast forms within granulomatous tissue (original magnification 100x). **(D)** Additional GMS stain confirming broad-based budding yeast, morphologically consistent with *Blastomyces dermatitidis* (original magnification 200x). **(E)** Fungal culture at 30 °C showing mold phase with septate hyphae and characteristic conidia.

Dermatology obtained incisional biopsies of the left anterior shin and left upper-arm nodules within 3 days of admission. Copious purulent and necrotic material was expressed from both sites. Histopathology of H&E-stained sections showed granulomatous inflammation with mixed acute and chronic infiltrates (Figures 3A,B).

Periodic acid–Schiff and Grocott methenamine silver stains revealed numerous large (8–15 μm) thick-walled yeast forms with broad-based budding (Figures 3C,D), morphologically consistent with *Blastomyces dermatitidis*.

Biopsy cultures on Sabouraud dextrose agar at 30 °C grew a white-tan, fluffy mold with a characteristic “lollipop” conidial arrangement, consistent with *Blastomyces*. The isolate was later identified as *Blastomyces dermatitidis/gilchristii* by matrix-assisted laser desorption/ionization time-of-flight mass spectrometry (MALDI-TOF MS) (Figure 3E). Internal transcribed spacer (ITS) sequencing was not performed because the isolate was identified as *Blastomyces dermatitidis* by MALDI-TOF MS in a CLIA-certified reference laboratory. When validated reference spectra are available, MALDI-TOF MS provides reliable species-level identification of *Blastomyces*.

Sensitivity testing using CLSI M38 broth microdilution was performed at the University of Texas Health Science Center at San Antonio Fungus Testing Laboratory.

Antifungal susceptibility testing demonstrated low minimum inhibitory concentrations to amphotericin B and triazole agents, with elevated fluconazole MICs; detailed results are summarized in Supplementary Table 3. These values fall within known susceptibility ranges for *Blastomyces* and are summarized in Supplementary Table 3. This pattern marked by very low MICs to amphotericin B and itraconazole and a high MIC to fluconazole is consistent with published susceptibility profiles (9–11) and supported the use of amphotericin B induction followed by itraconazole consolidation for the treatment of severe disseminated blastomycosis (1).

Fungal antigen testing results were as follows:

- Urine *Blastomyces* antigen: 11.08 U/mL (positive; ARUP assay).
- Serum *Blastomyces* antigen: 0.63 ng/mL (positive).
- Urine *Histoplasma* antigen: 5.20 ng/mL (positive).
- Serum *Histoplasma* antigen: 1.03 ng/mL (positive).
- Synovial fluid *Histoplasma* antigen: 1.76 ng/mL (positive).

The positive *Histoplasma* antigens detected in urine, serum, and synovial fluid were interpreted as false positives due to well-described antigenic cross-reactivity with *Blastomyces dermatitidis*, given histopathologic and culture confirmation of blastomycosis.

## Treatment and outcome

On hospital day 5, once biopsy confirmed disseminated blastomycosis, he received a 7-day course of liposomal amphotericin B 250 mg IV daily with pre- and post-infusion normal saline. Electrolytes were checked daily with proactive potassium and magnesium repletion; serum creatinine remained stable throughout. Liposomal amphotericin B was chosen over itraconazole as initial therapy because of disseminated disease, consistent with IDSA recommendations for moderate-to-severe disease (1). By day 3 of amphotericin therapy, knee pain had improved, fevers and sweats resolved, and the cutaneous nodules began to involute.

After 7 days of amphotericin, he transitioned to oral itraconazole 200 mg three times daily for 3 days, then 200 mg twice daily, with a planned duration of 6–12 months. Weekly complete blood count and complete metabolic panel monitoring were performed to detect potential amphotericin- or itraconazole-related nephrotoxicity, hepatotoxicity, or cytopenias during prolonged therapy. An itraconazole trough level was obtained at approximately 2 weeks, targeting ≥ 1.0 μg/mL. He received counseling on adherence and acid-suppressant interactions. The patient was discharged on hospital day 14 with infectious-diseases follow-up arranged.

At early follow-up, 7 weeks after discharge in Infectious Diseases clinic, he reported marked improvement: minimal residual knee discomfort without need for assistive devices, near-complete resolution of the skin lesions, no headache or neurological symptoms, no cough or constitutional symptoms, and a full return to work. He denied new adverse effects on itraconazole. Ongoing outpatient monitoring and dose adjustments were guided by clinical response, laboratory values, and drug levels. Serial urine Blastomyces antigen testing, which was initially positive at presentation, became undetectable after initiation of therapy, paralleling his clinical improvement. Itraconazole therapeutic drug monitoring demonstrated adequate serum levels within the recommended therapeutic range.

## Discussion

This case underscores the diagnostic complexity of inflammatory monoarthritis in an immunocompetent man ultimately diagnosed with disseminated blastomycosis. The presentation—synovial fluid revealing calcium pyrophosphate crystals and sterile bacterial cultures—initially pointed toward pseudogout. Short-lived improvement after intra-articular corticosteroid further obscured the underlying fungal etiology.

Blastomycosis rarely presents as monoarthritis. In our systematic literature review, fewer than 10 published case reports describing monoarthritis attributable to *Blastomyces dermatitidis* were identified (12). A recent report described a 78-year-old renal transplant recipient with chronic culture-negative monoarthritis of the ankle, initially misattributed to gout, who was ultimately diagnosed with *Blastomyces dermatitidis* septic arthritis by histopathology and antigen testing. Despite prolonged itraconazole therapy, the patient’s infection persisted, necessitating below-the-knee amputation, underscoring the potential for delayed diagnosis and poor outcomes in osteoarticular blastomycosis, particularly among immunosuppressed hosts (12). Bennie Ho et al. reported a 39-year-old immunocompetent man from southern Saskatchewan, Canada, who presented with disseminated *Blastomyces dermatitidis* infection, including septic arthritis of the right hip. The diagnosis was confirmed by growth of *B. dermatitidis* from urine and sputum cultures, with epididymal tissue histopathology showing broad-based budding yeast, highlighting that osteoarticular disease may be diagnosed from non-synovial specimens when joint fluid cultures are not obtained (6).

Musculoskeletal involvement more commonly reflects hematogenous dissemination or direct extension from osteomyelitis. In this case, the lung CT pattern of miliary infiltrates suggested a disseminated mycobacterial or fungal infection and the diagnosis was confirmed by biopsy of cutaneous nodules, revealing broad-based budding yeasts. Difficulty in obtaining the diagnosis resulted from: (1) low clinical suspicion of fungal disease in immunocompetent patients; (2) co-existing findings of calcium pyrophosphate crystal consistent with pseudogout; (3) overlapping clinical presentation with other granulomatous or fungal infections, and (4) a positive Histoplasma antigen, stemming from well-documented cross-reactivity between *Blastomyces dermatitidis* and *Histoplasma capsulatum* assays (13).

Disseminated blastomycosis can occur in otherwise healthy individuals, and risk factors in immunocompetent hosts include high-inoculum exposure, diabetes mellitus, smoking, or transient immune suppression such as corticosteroid use. The lungs are the most common primary site of infection, and in severe cases, pulmonary involvement can progress to acute respiratory distress syndrome (ARDS), which carries a high mortality rate. The patient’s prolonged respiratory symptoms predating his camping trip suggest exposure weeks to months earlier, consistent with the typical incubation period of 3 weeks to 3 months.

Although blastomycosis has been traditionally endemic in distinct regions—such as the Ohio and Mississippi River valleys, Great Lakes, and St. Lawrence Seaway—emerging evidence indicates expanding geographic distribution. Surveillance data show cases now span beyond historically defined boundaries, with outbreaks reported in areas previously considered nonendemic (2). Climate change is suspected to be contributing to these shifts, altering the environmental niche of fungal pathogens and facilitating their spread (14, 15). At our institution, from January 1, 2020, to the present, we have identified only eight culture-confirmed isolates of *Blastomyces dermatitidis* from six unique patients. Blastomycosis, while recognized as sporadically endemic within the state, particularly along the Susquehanna River Basin, is not a reportable condition in Pennsylvania. This is consistent with current public health practice in most US states, where *Blastomyces dermatitidis* infections are monitored through passive surveillance rather than mandatory reporting.

The diagnostic process in this case highlights several practical lessons. First, at least 3–5 mL of synovial fluid should be divided into aliquots for cell count, crystal analysis, and bacterial and fungal cultures; fungal PCR requires ≥ 0.5 mL, and AFB culture ≥ 2 mL. Second, clinicians should recognize that cross-reactivity between *Blastomyces* and *Histoplasma* antigens occurs in over 80% of cases (16). Antigen testing has a sensitivity of 70–90% in disseminated infection, highest in urine (17). Because of overlap, both assays should be considered in compatible presentations, with consideration for local endemicity and with confirmation by culture or histopathology. The (1 → 3)-*β*-D-glucan assay is typically negative in Blastomyces infection because its yeast form releases little circulating glucan. A practical diagnostic algorithm is as follows:

- Perform antigen testing for both Blastomyces and Histoplasma in compatible cases.
- Confirm with culture, histopathology, serology and/or molecular testing.
- Interpret positive *Blastomyces* or *Histoplasma* antigen with caution without correlating additional lab results.

The Infectious Diseases Society of America (IDSA) clinical practice guidelines stratify treatment of blastomycosis by disease severity and organ involvement (1). Mild-to-moderate non–life-threatening disease without central nervous system involvement is treated with oral itraconazole for 6–12 months, whereas moderate-to-severe or disseminated disease, including osteoarticular involvement, warrants initial induction therapy with amphotericin B. Liposomal amphotericin B is preferred because of improved tolerability, followed by step-down therapy with itraconazole once clinical improvement is achieved and cultures are controlled. Therapeutic drug monitoring is recommended during itraconazole therapy to ensure adequate serum concentrations (target trough ≥1.0 μg/mL) and to minimize toxicity. Fluconazole is considered less effective because of higher minimum inhibitory concentrations and is reserved for cases in which itraconazole cannot be used. This stepwise induction–consolidation approach was applied in our patient and resulted in rapid clinical improvement and sustained resolution of disseminated disease manifestations.

### Differential diagnosis

- Bacterial septic arthritis: Less likely given synovial WBC < 50,000/mm^3^, negative Gram stains, and sterile cultures.
- Sarcoidosis: Pulmonary nodules and skin lesions raised suspicion, but tissue biopsy with yeast forms excluded it.
- Histoplasmosis: Antigen positivity was misleading due to cross-reactivity.
- Coccidioidomycosis: Considered given the pulmonary and cutaneous involvement; however, epidemiologic exposure and confirmatory findings consistent with blastomycosis argued against this diagnosis.

### Lessons learned

1. Standard synovial analysis should always include cell count, crystals and culture. Unless fungal cultures or molecular assays are specifically requested, they may be overlooked.
2. Cutaneous biopsies are invaluable. Early dermatologic evaluation in disseminated illness can facilitate faster diagnosis.
3. Microbiology collaboration is critical, especially when handling dimorphic fungi requiring BSL-3 containment.
4. Fungal antigen cross-reactivity can be misleading. Positive Histoplasma results should not exclude blastomycosis—culture and histopathology remain definitive.

## Conclusion

In patients presenting with inflammatory monoarthritis, pulmonary infiltrates, and cutaneous lesions, disseminated blastomycosis should remain in the differential diagnosis—even in regions traditionally considered nonendemic. Awareness of host and environmental risk factors, including recent corticosteroid use and high-inoculum exposures, may help explain dissemination in immunocompetent patients. Ongoing changes in fungal geography underscore the need for multidisciplinary collaboration and early tissue biopsy. Recognizing cross-reactivity in antigen testing and ensuring appropriate diagnostic specimen allocation are key. Prompt antifungal therapy can lead to rapid, favorable clinical outcomes.

## Data Availability

The datasets presented in this article are not readily available because only anonymized clinical data relevant to the case (e.g., laboratory values, imaging summaries, and timelines) can be shared. No identifiable patient information or raw histopathology images containing protected health information will be released, in accordance with institutional and journal policies. Requests to access the datasets should be directed to Paddy Ssentongo, pssentongo@pennstatehealth.psu.edu.
