# Supplementary material for: Cutaneous clues to a fungal culprit: disseminated Blastomycosis presenting as inflammatory monoarthritis — a Case Report
Source: Front Med (Lausanne). 2026 Feb 18;13:1704751. doi: 10.3389/fmed.2026.1704751 (PMC12958069; doi:10.3389/fmed.2026.1704751)
Supplement: Supplementary file 1 [file Table_1.DOCX]

**Cutaneous Clues to a Fungal Culprit: Disseminated Blastomycosis Presenting as Monoarthritis: A Case Report**

| Section | Content | Reported on (Page) |
| --- | --- | --- |
| Title | Reported: Yes. Title contains diagnosis and 'case report.' | Page 1 |
| Key Words | Blastomycosis; Monoarthritis; Osteoarticular infection; Fungal infection; Case report | Page 1 |
| Abstract | Introduction (unique aspects): Rare presentation of blastomycosis as inflammatory monoarthritis in an immunocompetent host; adds to limited literature. Main symptoms/clinical findings: Knee swelling and pain, nodular skin lesions, cough, pulmonary nodules. Main diagnoses/interventions/outcomes: Initially diagnosed as pseudogout → ultimately disseminated Blastomyces dermatitidis confirmed by biopsy/culture. Treated with amphotericin B then itraconazole → rapid improvement. Conclusion/take-away: Fungal infection should remain in differential of monoarthritis; importance of multidisciplinary evaluation and tissue biopsy. | Page 1 |
| Introduction | Reported: Yes, with background on epidemiology, rarity of osteoarticular involvement, diagnostic challenges. | Page 2 |
| Patient Information | De-identified patient data: 41-year-old previously healthy man. Primary concerns/symptoms: Left knee pain and swelling, nodular skin lesions, chronic cough, intermittent fevers/night sweats. Medical/family/psychosocial history: No relevant PMH; immunocompetent. Relevant past interventions/outcomes: Treated as pseudogout (colchicine, cephalexin), intra-articular corticosteroid, oral clindamycin—no durable response. | Pages 2–3 |
| Clinical Findings | Physical exam: Diffusely swollen/tender left knee with effusion; multiple cutaneous nodules (shoulder, shin, arm, flank). | Page 3 |
| Timeline | Reported: Yes. Detailed timeline from Day –42 (symptom onset after camping) through admission (Day 0), investigations, interventions, and follow-up. | Pages 2–4 |
| Diagnostic Assessment | Tests: Synovial fluid analyses, bacterial cultures, fungal antigen assays, CT chest/abdomen/pelvis, MRI brain, dermatology biopsy with PAS/GMS staining, fungal cultures. Diagnostic challenges: Misleading CPPD crystals, false-positive Histoplasma antigen, immunocompetent status leading to low suspicion. Diagnosis: Disseminated Blastomyces dermatitidis (confirmed by culture, histopathology, antigen positivity). Prognosis: Favorable with antifungal therapy; no CNS involvement established. | Pages 3–5 |
| Therapeutic Intervention | Types: Pharmacologic—liposomal amphotericin B → itraconazole step-down. Administration: Amphotericin B 250 mg IV daily × 7 days; transitioned to itraconazole 200 mg PO TID × 3 days, then BID, planned 6–12 months. Changes/rationale: Step-down once culture confirmed blastomycosis and patient clinically improved. | Page 5 |
| Follow-up and Outcomes | Clinician/patient-assessed outcomes: Resolution of fevers, knee pain, cutaneous lesions; regained ambulation. Follow-up tests: Weekly labs, itraconazole level monitoring. Adherence/tolerability: Good; no adverse effects reported. Adverse events: None unexpected; stable renal function on amphotericin. | Pages 5–6 |
| Discussion | Strengths/limitations: Multidisciplinary approach, culture confirmation; limitation—diagnostic delay due to misleading crystals/antigen cross-reactivity. Relevant literature: Compared with <10 prior reports of blastomycosis presenting as monoarthritis; discussed misdiagnosis pitfalls and antigen cross-reactivity. Rationale for conclusions: Culture, histopathology, and imaging evidence. Primary take-away lessons: Consider fungal etiologies in atypical monoarthritis; tissue biopsy critical; beware of antigen cross-reactivity. | Pages 6–8 |
| Patient Perspective | Not reported. | N/A |
| Informed Consent | Reported: Yes—written consent obtained. | Page 18 |
