# Supplementary material for: Cutaneous clues to a fungal culprit: disseminated Blastomycosis presenting as inflammatory monoarthritis — a Case Report
Source: Front Med (Lausanne). 2026 Feb 18;13:1704751. doi: 10.3389/fmed.2026.1704751 (PMC12958069; doi:10.3389/fmed.2026.1704751)
Supplement: Supplementary file 2 [file Table_2.DOCX]

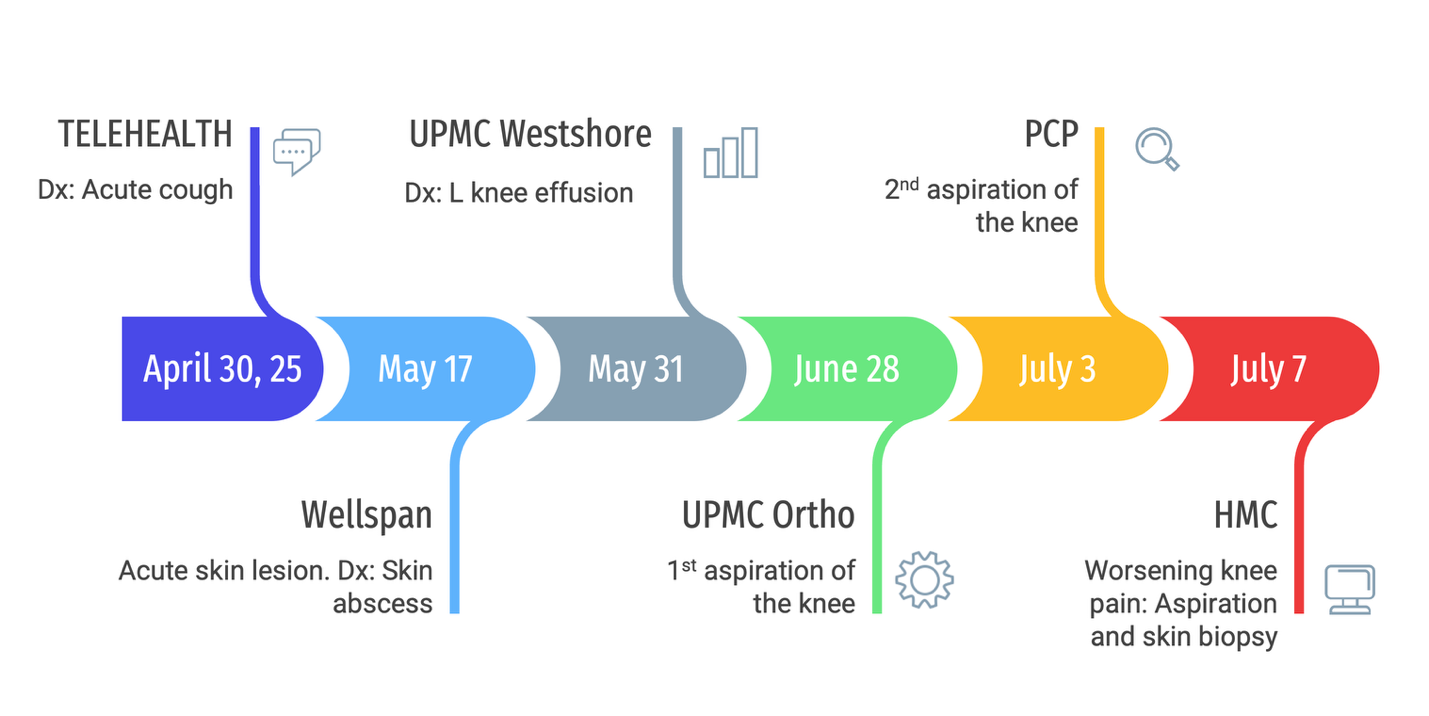


**Supplementary Figure 1.** Timeline of diagnostic evaluations and interventions prior to definitive diagnosis. The patient underwent multiple evaluations for persistent knee pain, cutaneous lesions, and cough across several health systems, with initial diagnoses including acute cough, skin abscess, and pseudogout. Repeated knee aspirations and empirical antibacterial therapy provided only transient or no relief. The final diagnosis of disseminated *Blastomyces dermatitidis*was established after biopsy and fungal culture at our institution (HMC).
