# Supplementary material for: Cutaneous clues to a fungal culprit: disseminated Blastomycosis presenting as inflammatory monoarthritis — a Case Report
Source: Front Med (Lausanne). 2026 Feb 18;13:1704751. doi: 10.3389/fmed.2026.1704751 (PMC12958069; doi:10.3389/fmed.2026.1704751)
Supplement: Supplementary file 3 [file Table_3.DOCX]

| **Variable** | **Reference Range** | **On Admission** |
| --- | --- | --- |
| White-cell count (per µl) | 4,500–10,000 | 20,290 ↑ |
| Neutrophils (%) | 40–70 | 70.3 |
| Lymphocytes (%) | 20–45 | 18.9 |
| Monocytes (%) | 2–10 | 10.8 |
| Eosinophils (%) | 0–6 | 0.0 |
| Basophils (%) | 0–2 | 0.0 |
| Hemoglobin (g/dl) | 13.5–17.5 | 14.2 |
| Hematocrit (%) | 41–53 | 43.4 |
| Red-cell count (million/µl) | 4.5–5.9 | 5.10 |
| MCV (fl) | 80–96 | 85.1 |
| MCHC (g/dl) | 32–36 | 32.7 |
| MCH (pg) | 27–33 | 27.8 ↓ |
| Platelet count (per µl) | 150,000–400,000 | 535,000 ↑ |
| Sodium (mmol/liter) | 135–145 | 139 |
| Potassium (mmol/liter) | 3.4–5.0 | 4.4 |
| Chloride (mmol/liter) | 98–108 | 100 |
| HC03 (mmol/liter) | 23–32 | 23 |
| Anion gap | 3–11 | 16 ↑ |
| Urea nitrogen (mg/dl) | 8–25 | 11 |
| Creatinine (mg/dl) | 0.60–1.50 | 0.86 |
| Estimated CrCl (ml/min) | — | 118.22 |
| eGFR CKD-EPI (ml/min/1.73 m²) | ≥60 | >90 |
| Glucose (mg/dl) | 70–110 | 106* |
| Calcium (mg/dl) | 8.5–10.5 | 9.8 |
| Aspartate aminotransferase (U/liter) | 10–40 | 26 |
| Alanine aminotransferase (U/liter) | 10–55 | 67 ↑ |
| Alkaline phosphatase (U/liter) | 45–115 | 83 |
| Total bilirubin (mg/dl) | 0.0–1.0 | 0.3 |
| Albumin (g/dl) | 3.3–5.0 | 4.0 |
| Total protein (g/dl) | 6.0–8.3 | 8.3 |
| Vitamin D, 25-hydroxy (ng/ml) | 30–100 | 35 |
| Rheumatoid factor (IU/ml) | <14 | 22.88 ↑ |
| Erythrocyte sedimentation rate (mm/hr) | 0–13 | 108 ↑ |
| Lyme antibodies, IgG/IgM | Negative | Negative |
| RPR | Nonreactive | Nonreactive |
| Fungitell (1→3)-β-D-glucan (pg/ml) | <31 | <31* |
| HIV Ag/Ab | Negative | Negative |

**Supplementary Table 1. Laboratory Values on Admission.** Values outside the reference range are indicated with arrows (↑ above normal, ↓ below normal). * denotes borderline or near-threshold values. CrCl denotes creatinine clearance; eGFR CKD-EPI denotes estimated glomerular filtration rate calculated with the CKD-EPI equation.

| **Setting** | **WBC/mm³** | **Differential** | **Crystals** | **Culture** |
| --- | --- | --- | --- | --- |
| Outside facility (≈5 weeks prior) | ~13,000 | Neutrophil‑predominant | Calcium pyrophosphate | Rare coagulase‑negative *Staphylococcus* (contaminant) |
| Our medical center (day of admission) | 6,000 | 92% neutrophils, 7% lymphocytes, 1% monocytes/macrophages | None | No growth |

**Supplementary Table 2. Synovial Fluid Analyses.** Findings from arthrocentesis performed at an outside facility approximately 5 weeks prior to admission and at our medical center on the day of admission. WBC denotes white blood cell count.

| Antifungal | Patient Isolate MIC (µg/mL) | MIC Range^1-3^ |
| --- | --- | --- |
| Amphotericin B | ≤0.03 | <0.03 – 2.0 |
| Fluconazole | 32 | 0.06 – 32 |
| Itraconazole | 0.06 | <0.01 – 0.13 |
| Voriconazole | 0.25 | <0.03 – 0.25 |
| Posaconazole | 0.125 | <0.02 – 0.06 |
| Isavuconazole | 0.25 | 0.125–0.5 |

**Supplementary Table 3 Antifungal Susceptibility Profile of the Patient’s Blastomyces Isolate Compared With Published MIC Ranges.** The table summarizes minimum inhibitory concentrations (MICs) obtained from the patient’s isolate and compares them with published yeast-phase MIC ranges and mold-phase ranges

**References**

1. Dukik K, Al-Hatmi AM, Curfs-Breuker I, Faro D, De Hoog S, Meis JF. Antifungal susceptibility of emerging dimorphic pathogens in the family Ajellomycetaceae. *Antimicrobial agents and chemotherapy.* 2018;62(1):10.1128/aac. 01886-01817.

2. Lewis JS, Wiederhold NP, Hakki M, Thompson III GR. New perspectives on antimicrobial agents: isavuconazole. *Antimicrobial agents and chemotherapy.* 2022;66(9):e00177-00122.

3. Goughenour KD, Rappleye CA. Antifungal therapeutics for dimorphic fungal pathogens. *Virulence.* 2017;8(2):211-221.
